# *N*,*N*,*N*′,*N*′-Tetra­ethyl­pyridine-2,6-dicarboxamide

**DOI:** 10.1107/S1600536811045727

**Published:** 2011-11-05

**Authors:** Michaela Pojarová, Michal Dušek, Emanuel Makrlík, Vasily A. Babain

**Affiliations:** a Institute of Physics, AS CR, v.v.i., Na Slovance 2, 182 21 Praha 8, Czech Republic; b Faculty of Environmetal Sciences, Czech University of Life Sciences, Prague, Kamýcká 129, 165 21 Prague 6, Czech Republic; c Khlopin Radium Institute, Research and Production Association, 2nd Murinskiy Prospect b. 28, 194021 St. Petersburg, Russian Federation

## Abstract

The title compound, C_15_H_23_N_3_O_2_, crystallizes with two mol­ecules in the asymmetric unit which are linked by a C—H⋯N hydrogen bond. In the crystal, mol­ecules are connected *via* weak C—H⋯O and C—H⋯N hydrogen bonds between the amide O atoms and ethyl chains and between pyridine N atoms and aromatic H atoms in *para* positions. C—H⋯π inter­actions also occur.

## Related literature

The title compound has been investigated for its extractive properties in a synergistic mixture with chlorinated cobalt dicarbollide towards trivalent metals, see: Alyapyshev *et al.* (2004 ▶). For details of the synthesis, see: Nikitskaya *et al.* (1958 ▶); Shimada *et al.* (2004 ▶).
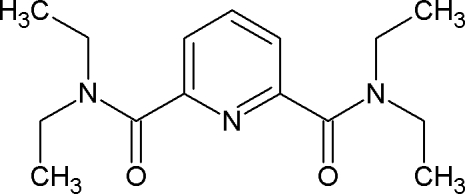


## Experimental

### 

#### Crystal data

C_15_H_23_N_3_O_2_
*M* *_r_* = 277.36
Triclinic, 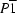

*a* = 11.1919 (3) Å
*b* = 11.7913 (3) Å
*c* = 12.2774 (3) Å
α = 90.255 (2)°
β = 105.050 (2)°
γ = 102.600 (2)°
*V* = 1523.74 (7) Å^3^
*Z* = 4
Cu *K*α radiation
μ = 0.65 mm^−1^
*T* = 120 K
0.53 × 0.38 × 0.14 mm

#### Data collection

Agilent Xcalibur Atlas Gemini ultra diffractometer
Absorption correction: analytical (*CrysAlis PRO*; Agilent, 2011 ▶) *T*_min_ = 0.538, *T*_max_ = 0.815
17964 measured reflections
5424 independent reflections
5034 reflections with *I* > 2σ(*I*)
*R*_int_ = 0.023

#### Refinement

*R*[*F* ^2^ > 2σ(*F* ^2^)] = 0.033
*wR*(*F* ^2^) = 0.089
*S* = 1.04
5424 reflections
369 parameters
H-atom parameters constrained
Δρ_max_ = 0.24 e Å^−3^
Δρ_min_ = −0.23 e Å^−3^

### 

Data collection: *CrysAlis PRO* (Agilent, 2011 ▶); cell refinement: *CrysAlis PRO*; data reduction: *CrysAlis PRO*; program(s) used to solve structure: *SHELXS97* (Sheldrick, 2008 ▶); program(s) used to refine structure: *SHELXL97* (Sheldrick, 2008 ▶); molecular graphics: *Mercury* (Macrae *et al.*, 2006 ▶) and *DIAMOND* (Brandenburg & Putz, 2005 ▶); software used to prepare material for publication: *publCIF* (Westrip, 2010 ▶).

## Supplementary Material

Crystal structure: contains datablock(s) I, global. DOI: 10.1107/S1600536811045727/hg5112sup1.cif

Structure factors: contains datablock(s) I. DOI: 10.1107/S1600536811045727/hg5112Isup2.hkl

Supplementary material file. DOI: 10.1107/S1600536811045727/hg5112Isup3.cml

Additional supplementary materials:  crystallographic information; 3D view; checkCIF report

## Figures and Tables

**Table 1:** Hydrogen-bond geometry (Å, °) *Cg*2 is the centroid of the N4/C16–C20 ring.

*D*—H⋯*A*	*D*—H	H⋯*A*	*D*⋯*A*	*D*—H⋯*A*
C3—H3⋯N4^i^	0.93	2.49	3.397 (1)	164
C9—H9*B*⋯O4^ii^	0.97	2.52	3.485 (1)	175
C12—H12*B*⋯O3^ii^	0.97	2.59	3.531 (1)	164
C18—H18⋯N1	0.93	2.52	3.427 (1)	166
C24—H24*A*⋯O2^iii^	0.97	2.46	3.417 (1)	169
C27—H27*A*⋯O1^iii^	0.97	2.54	3.496 (1)	168
C13—H13*B*⋯*Cg*(2)^ii^	0.97	2.97	3.726 (1)	139

Symmetry codes: (i) 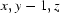; (ii) 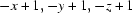; (iii) 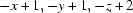.

**Fig. 1.**
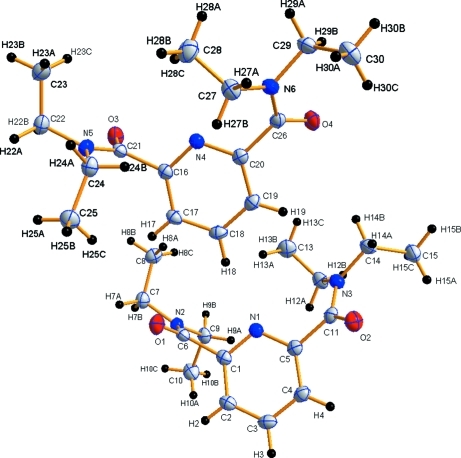
View of the asymmetric unit of N,N,N',N'-tetraethyl-2,6-dipicolinamide, together with atom-labelling scheme. Displacement ellipsoids are shown at the 50% probability level.

**Fig. 2.**
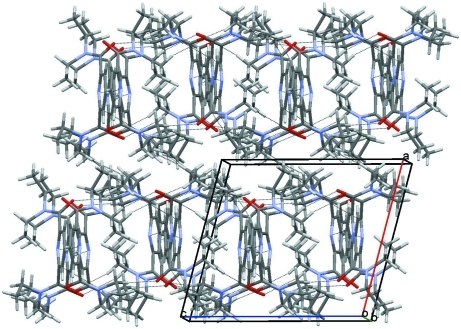
Projection along the b axis with highlighted hydrogen bonds between the molecules in the bands in direction of c axis.

## supplementary crystallographic information

### Comment

The title compound, shown in Figure 1 and Scheme 1, has been investigated in mixture with the dicarbollylcobaltate anion and its halogen derivatives for significant extraction properties towards trivalent metal cations (Alyapyshev *et al.*,2004). It consists of pyridine ring with a diethylamide groups in position 2 and 6. The asymmetric unit contains two molecules of dipicolinamide connected *via* hydrogen bonds between pyridine nitrogen atom and aromatic hydrogen atoms in *para* position to nitrogen atoms (C3—H3···N4, and C18—H18···N1, Table 1). While at first impression, the amide groups seem to be related by a mirror plane, closer look reveals their differences. The carbon atoms of carbonyl groups do not lie in a plane of the pyridine ring and they differ in the distance to this plane (0.178 Å for C6 and 0.089 Å for C11 to pyridine plane with N1; 0.146 Å for C21 and 0.040 Å for C26 to pyridine plane with N4). The molecules form bands in direction of the *c* axis (Fig. 2) *via* system of hydrogen bonds between the amide oxygen atom and ethyl chains (Table 1).

### Experimental

*N*,*N*,*N*',*N*'-Tetraethyl-2,6-dipicolinamide was synthesized as described in Shimada *et al.* (2004), and Nikitskaya *et al.* (1958). Crystals were prepared by slow evaporation from acetonitrile.

### Refinement

The hydrogen atoms were localized from the difference Fourier map. Despite that, all hydrogen atoms connected to C were constrained to ideal positions with C—H = 0.93 - 0.97Å. The isotropic temperature parameters of hydrogen atoms were calculated as 1.2**U*_eq_ of the parent atom.

### Crystal data

**Table:**

C_15_H_23_N_3_O_2_	*Z* = 4
*M**_r_* = 277.36	*F*(000) = 600
Triclinic, *P*1	*D*_x_ = 1.209 Mg m^−^^3^
Hall symbol: -P 1	Cu *K*α radiation, λ = 1.5418 Å
*a* = 11.1919 (3) Å	Cell parameters from 10531 reflections
*b* = 11.7913 (3) Å	θ = 3.7–66.9°
*c* = 12.2774 (3) Å	µ = 0.65 mm^−^^1^
α = 90.255 (2)°	*T* = 120 K
β = 105.050 (2)°	Prism, colourless
γ = 102.600 (2)°	0.53 × 0.38 × 0.14 mm
*V* = 1523.74 (7) Å^3^	

### Data collection

**Table:**

Agilent Xcalibur Atlas Gemini ultra diffractometer	5424 independent reflections
Radiation source: Enhance Ultra (Cu) X-ray Source	5034 reflections with *I* > 2σ(*I*)
mirror	*R*_int_ = 0.023
Detector resolution: 10.3784 pixels mm^-1^	θ_max_ = 67.0°, θ_min_ = 3.7°
Rotation method data acquisition using ω scans	*h* = −13→13
Absorption correction: analytical (*CrysAlis PRO*; Agilent, 2011)	*k* = −14→14
*T*_min_ = 0.538, *T*_max_ = 0.815	*l* = −14→13
17964 measured reflections	

### Refinement

**Table:**

Refinement on *F*^2^	Primary atom site location: structure-invariant direct methods
Least-squares matrix: full	Secondary atom site location: difference Fourier map
*R*[*F*^2^ > 2σ(*F*^2^)] = 0.033	Hydrogen site location: inferred from neighbouring sites
*wR*(*F*^2^) = 0.089	H-atom parameters constrained
*S* = 1.04	*w* = 1/[σ^2^(*F*_o_^2^) + (0.0492*P*)^2^ + 0.3468*P*] where *P* = (*F*_o_^2^ + 2*F*_c_^2^)/3
5424 reflections	(Δ/σ)_max_ < 0.001
369 parameters	Δρ_max_ = 0.24 e Å^−^^3^
0 restraints	Δρ_min_ = −0.23 e Å^−^^3^

### Special details

**Table:**

Geometry. All e.s.d.'s (except the e.s.d. in the dihedral angle between two l.s. planes) are estimated using the full covariance matrix. The cell e.s.d.'s are taken into account individually in the estimation of e.s.d.'s in distances, angles and torsion angles; correlations between e.s.d.'s in cell parameters are only used when they are defined by crystal symmetry. An approximate (isotropic) treatment of cell e.s.d.'s is used for estimating e.s.d.'s involving l.s. planes.
Refinement. Refinement of *F*^2^ against ALL reflections. The weighted *R*-factor *wR* and goodness of fit *S* are based on *F*^2^, conventional *R*-factors *R* are based on *F*, with *F* set to zero for negative *F*^2^. The threshold expression of *F*^2^ > σ(*F*^2^) is used only for calculating *R*-factors(gt) *etc*. and is not relevant to the choice of reflections for refinement. *R*-factors based on *F*^2^ are statistically about twice as large as those based on *F*, and *R*- factors based on ALL data will be even larger. The hydrogen atoms were localized from the difference Fourier map. Despite of that, all hydrogen atoms connected to C were constrained to ideal positions with C—H = 0.93 - 0.97Å. The isotropic temperature parameters of hydrogen atoms were calculated as 1.2**U*_eq_ of the parent atom.

### Fractional atomic coordinates and isotropic or equivalent isotropic displacement parameters (Å^2^)

**Table:**

	*x*	*y*	*z*	*U*_iso_*/*U*_eq_	
C1	0.34518 (10)	0.08087 (9)	0.64634 (8)	0.0204 (2)	
C2	0.33423 (11)	−0.03673 (9)	0.66482 (9)	0.0246 (2)	
H2	0.2551	−0.0882	0.6484	0.030*	
C3	0.44453 (11)	−0.07514 (9)	0.70844 (9)	0.0269 (2)	
H3	0.4405	−0.1533	0.7218	0.032*	
C4	0.56088 (11)	0.00389 (9)	0.73192 (9)	0.0243 (2)	
H4	0.6358	−0.0196	0.7637	0.029*	
C5	0.56290 (10)	0.11901 (9)	0.70686 (8)	0.0203 (2)	
C6	0.23248 (10)	0.13636 (9)	0.61227 (9)	0.0211 (2)	
C7	0.08199 (10)	0.20920 (10)	0.46756 (10)	0.0263 (2)	
H7A	0.0343	0.2047	0.5237	0.032*	
H7B	0.0235	0.1753	0.3962	0.032*	
C8	0.13952 (11)	0.33599 (10)	0.45498 (10)	0.0294 (2)	
H8A	0.1995	0.3690	0.5248	0.035*	
H8B	0.0734	0.3782	0.4362	0.035*	
H8C	0.1820	0.3409	0.3960	0.035*	
C9	0.22324 (10)	0.09539 (10)	0.41142 (9)	0.0245 (2)	
H9A	0.3102	0.0880	0.4413	0.029*	
H9B	0.2217	0.1492	0.3518	0.029*	
C10	0.14003 (12)	−0.02268 (11)	0.36241 (11)	0.0343 (3)	
H10A	0.1516	−0.0791	0.4181	0.041*	
H10B	0.1632	−0.0459	0.2971	0.041*	
H10C	0.0526	−0.0178	0.3410	0.041*	
C11	0.68695 (10)	0.20972 (9)	0.73695 (9)	0.0213 (2)	
C12	0.63999 (11)	0.27551 (10)	0.54038 (9)	0.0264 (2)	
H12A	0.5875	0.1971	0.5229	0.032*	
H12B	0.7009	0.2854	0.4956	0.032*	
C13	0.55615 (12)	0.36185 (12)	0.50620 (11)	0.0357 (3)	
H13A	0.4917	0.3492	0.5462	0.043*	
H13B	0.5168	0.3512	0.4263	0.043*	
H13C	0.6070	0.4398	0.5244	0.043*	
C14	0.82160 (11)	0.38506 (10)	0.69519 (10)	0.0282 (2)	
H14A	0.8443	0.3990	0.7767	0.034*	
H14B	0.8001	0.4549	0.6619	0.034*	
C15	0.93595 (11)	0.36358 (11)	0.66058 (12)	0.0344 (3)	
H15A	0.9613	0.2974	0.6969	0.041*	
H15B	1.0048	0.4310	0.6829	0.041*	
H15C	0.9141	0.3488	0.5801	0.041*	
N1	0.45723 (8)	0.15775 (7)	0.66542 (7)	0.01987 (19)	
N2	0.18053 (8)	0.14265 (8)	0.50181 (7)	0.0226 (2)	
N3	0.70930 (8)	0.28815 (8)	0.66088 (8)	0.0229 (2)	
O1	0.19638 (7)	0.17751 (7)	0.68735 (7)	0.02933 (19)	
O2	0.76002 (8)	0.21063 (7)	0.83144 (6)	0.02971 (19)	
C16	0.34289 (10)	0.58314 (9)	0.77594 (8)	0.0194 (2)	
C17	0.33178 (10)	0.46562 (9)	0.75081 (9)	0.0226 (2)	
H17	0.2525	0.4144	0.7285	0.027*	
C18	0.44214 (11)	0.42685 (9)	0.75996 (9)	0.0243 (2)	
H18	0.4381	0.3489	0.7431	0.029*	
C19	0.55846 (10)	0.50556 (9)	0.79455 (9)	0.0227 (2)	
H19	0.6336	0.4820	0.7991	0.027*	
C20	0.56057 (10)	0.62050 (9)	0.82237 (8)	0.0192 (2)	
C21	0.22925 (9)	0.63767 (9)	0.75813 (9)	0.0199 (2)	
C22	0.07365 (10)	0.70041 (10)	0.83483 (10)	0.0259 (2)	
H22A	0.0197	0.6636	0.8810	0.031*	
H22B	0.0230	0.6910	0.7568	0.031*	
C23	0.12039 (12)	0.82907 (10)	0.87084 (11)	0.0326 (3)	
H23A	0.1691	0.8386	0.9485	0.039*	
H23B	0.0490	0.8641	0.8624	0.039*	
H23C	0.1727	0.8660	0.8244	0.039*	
C24	0.22581 (10)	0.59551 (10)	0.95535 (9)	0.0240 (2)	
H24A	0.2183	0.6452	1.0152	0.029*	
H24B	0.3150	0.5956	0.9675	0.029*	
C25	0.15180 (11)	0.47232 (10)	0.96115 (10)	0.0295 (3)	
H25A	0.0624	0.4697	0.9379	0.035*	
H25B	0.1743	0.4493	1.0373	0.035*	
H25C	0.1719	0.4202	0.9119	0.035*	
C26	0.68515 (10)	0.70986 (9)	0.85781 (9)	0.0201 (2)	
C27	0.62809 (10)	0.77044 (10)	1.02959 (9)	0.0256 (2)	
H27A	0.6847	0.7776	1.1052	0.031*	
H27B	0.5737	0.6927	1.0177	0.031*	
C28	0.54606 (12)	0.85895 (12)	1.02167 (11)	0.0358 (3)	
H28A	0.5992	0.9362	1.0334	0.043*	
H28B	0.5003	0.8466	1.0784	0.043*	
H28C	0.4869	0.8500	0.9482	0.043*	
C29	0.81996 (11)	0.87818 (10)	0.97545 (10)	0.0279 (2)	
H29A	0.8002	0.9477	1.0020	0.034*	
H29B	0.8475	0.8958	0.9075	0.034*	
C30	0.92803 (11)	0.84799 (11)	1.06496 (10)	0.0318 (3)	
H30A	0.9023	0.8325	1.1332	0.038*	
H30B	1.0008	0.9122	1.0797	0.038*	
H30C	0.9494	0.7802	1.0387	0.038*	
N4	0.45478 (8)	0.65964 (7)	0.81247 (7)	0.01912 (18)	
N5	0.17952 (8)	0.64264 (8)	0.84598 (7)	0.02175 (19)	
N6	0.70426 (8)	0.78454 (8)	0.94712 (7)	0.0219 (2)	
O3	0.18965 (7)	0.67712 (7)	0.66658 (6)	0.02781 (18)	
O4	0.76290 (7)	0.71225 (7)	0.80198 (7)	0.02781 (18)	

### Atomic displacement parameters (Å^2^)

**Table:**

	*U*^11^	*U*^22^	*U*^33^	*U*^12^	*U*^13^	*U*^23^
C1	0.0241 (5)	0.0214 (5)	0.0163 (5)	0.0032 (4)	0.0081 (4)	0.0002 (4)
C2	0.0278 (6)	0.0213 (5)	0.0241 (5)	0.0010 (4)	0.0098 (4)	0.0018 (4)
C3	0.0376 (6)	0.0189 (5)	0.0270 (6)	0.0070 (4)	0.0133 (5)	0.0052 (4)
C4	0.0290 (6)	0.0251 (5)	0.0230 (5)	0.0111 (4)	0.0100 (4)	0.0053 (4)
C5	0.0241 (5)	0.0224 (5)	0.0165 (5)	0.0064 (4)	0.0083 (4)	0.0009 (4)
C6	0.0210 (5)	0.0185 (5)	0.0227 (5)	−0.0007 (4)	0.0083 (4)	0.0002 (4)
C7	0.0235 (5)	0.0312 (6)	0.0254 (5)	0.0083 (4)	0.0067 (4)	0.0032 (4)
C8	0.0324 (6)	0.0287 (6)	0.0262 (6)	0.0089 (5)	0.0048 (5)	0.0022 (4)
C9	0.0258 (5)	0.0292 (6)	0.0206 (5)	0.0063 (4)	0.0098 (4)	0.0019 (4)
C10	0.0350 (6)	0.0342 (6)	0.0333 (6)	0.0032 (5)	0.0127 (5)	−0.0066 (5)
C11	0.0221 (5)	0.0226 (5)	0.0214 (5)	0.0078 (4)	0.0077 (4)	−0.0012 (4)
C12	0.0272 (6)	0.0311 (6)	0.0216 (5)	0.0029 (4)	0.0105 (4)	0.0028 (4)
C13	0.0374 (7)	0.0403 (7)	0.0306 (6)	0.0104 (5)	0.0101 (5)	0.0123 (5)
C14	0.0261 (6)	0.0228 (5)	0.0348 (6)	0.0007 (4)	0.0107 (5)	−0.0021 (5)
C15	0.0237 (6)	0.0341 (6)	0.0444 (7)	0.0020 (5)	0.0113 (5)	−0.0022 (5)
N1	0.0224 (4)	0.0198 (4)	0.0183 (4)	0.0045 (3)	0.0074 (3)	0.0010 (3)
N2	0.0223 (4)	0.0248 (5)	0.0224 (4)	0.0060 (4)	0.0085 (4)	0.0019 (4)
N3	0.0223 (4)	0.0222 (4)	0.0240 (5)	0.0022 (4)	0.0083 (4)	−0.0001 (4)
O1	0.0282 (4)	0.0382 (5)	0.0241 (4)	0.0096 (3)	0.0098 (3)	−0.0030 (3)
O2	0.0281 (4)	0.0343 (4)	0.0237 (4)	0.0062 (3)	0.0022 (3)	0.0003 (3)
C16	0.0215 (5)	0.0209 (5)	0.0160 (5)	0.0032 (4)	0.0068 (4)	0.0018 (4)
C17	0.0241 (5)	0.0210 (5)	0.0210 (5)	0.0007 (4)	0.0068 (4)	−0.0013 (4)
C18	0.0323 (6)	0.0185 (5)	0.0233 (5)	0.0060 (4)	0.0092 (5)	−0.0012 (4)
C19	0.0250 (5)	0.0243 (5)	0.0211 (5)	0.0088 (4)	0.0075 (4)	0.0011 (4)
C20	0.0223 (5)	0.0211 (5)	0.0155 (5)	0.0052 (4)	0.0069 (4)	0.0027 (4)
C21	0.0189 (5)	0.0175 (5)	0.0211 (5)	0.0002 (4)	0.0052 (4)	−0.0003 (4)
C22	0.0235 (5)	0.0312 (6)	0.0270 (6)	0.0107 (4)	0.0097 (4)	0.0040 (4)
C23	0.0401 (7)	0.0298 (6)	0.0355 (6)	0.0147 (5)	0.0177 (5)	0.0055 (5)
C24	0.0245 (5)	0.0300 (6)	0.0191 (5)	0.0084 (4)	0.0069 (4)	0.0025 (4)
C25	0.0321 (6)	0.0316 (6)	0.0275 (6)	0.0095 (5)	0.0105 (5)	0.0076 (5)
C26	0.0204 (5)	0.0203 (5)	0.0207 (5)	0.0065 (4)	0.0059 (4)	0.0048 (4)
C27	0.0255 (5)	0.0301 (6)	0.0200 (5)	0.0025 (4)	0.0074 (4)	−0.0014 (4)
C28	0.0366 (7)	0.0418 (7)	0.0326 (6)	0.0125 (5)	0.0124 (5)	−0.0073 (5)
C29	0.0269 (6)	0.0210 (5)	0.0323 (6)	−0.0016 (4)	0.0075 (5)	−0.0004 (4)
C30	0.0247 (6)	0.0346 (6)	0.0306 (6)	−0.0031 (5)	0.0057 (5)	0.0005 (5)
N4	0.0205 (4)	0.0197 (4)	0.0177 (4)	0.0042 (3)	0.0062 (3)	0.0021 (3)
N5	0.0217 (4)	0.0238 (4)	0.0214 (4)	0.0069 (3)	0.0071 (4)	0.0022 (3)
N6	0.0216 (4)	0.0205 (4)	0.0225 (4)	0.0014 (3)	0.0070 (4)	0.0007 (3)
O3	0.0256 (4)	0.0365 (4)	0.0233 (4)	0.0096 (3)	0.0076 (3)	0.0083 (3)
O4	0.0248 (4)	0.0331 (4)	0.0282 (4)	0.0045 (3)	0.0135 (3)	0.0022 (3)

### Geometric parameters (Å, °)

**Table:**

C1—N1	1.3412 (13)	C16—N4	1.3397 (13)
C1—C2	1.3900 (15)	C16—C17	1.3900 (15)
C1—C6	1.5108 (15)	C16—C21	1.5141 (14)
C2—C3	1.3851 (17)	C17—C18	1.3864 (16)
C2—H2	0.9300	C17—H17	0.9300
C3—C4	1.3857 (16)	C18—C19	1.3852 (16)
C3—H3	0.9300	C18—H18	0.9300
C4—C5	1.3899 (15)	C19—C20	1.3899 (15)
C4—H4	0.9300	C19—H19	0.9300
C5—N1	1.3383 (14)	C20—N4	1.3393 (14)
C5—C11	1.5134 (14)	C20—C26	1.5112 (14)
C6—O1	1.2342 (13)	C21—O3	1.2296 (13)
C6—N2	1.3390 (14)	C21—N5	1.3424 (14)
C7—N2	1.4658 (14)	C22—N5	1.4682 (14)
C7—C8	1.5190 (16)	C22—C23	1.5160 (17)
C7—H7A	0.9700	C22—H22A	0.9700
C7—H7B	0.9700	C22—H22B	0.9700
C8—H8A	0.9600	C23—H23A	0.9600
C8—H8B	0.9600	C23—H23B	0.9600
C8—H8C	0.9600	C23—H23C	0.9600
C9—N2	1.4686 (14)	C24—N5	1.4667 (14)
C9—C10	1.5177 (16)	C24—C25	1.5191 (16)
C9—H9A	0.9700	C24—H24A	0.9700
C9—H9B	0.9700	C24—H24B	0.9700
C10—H10A	0.9600	C25—H25A	0.9600
C10—H10B	0.9600	C25—H25B	0.9600
C10—H10C	0.9600	C25—H25C	0.9600
C11—O2	1.2320 (13)	C26—O4	1.2357 (13)
C11—N3	1.3484 (14)	C26—N6	1.3457 (14)
C12—N3	1.4711 (14)	C27—N6	1.4722 (14)
C12—C13	1.5195 (17)	C27—C28	1.5213 (17)
C12—H12A	0.9700	C27—H27A	0.9700
C12—H12B	0.9700	C27—H27B	0.9700
C13—H13A	0.9600	C28—H28A	0.9600
C13—H13B	0.9600	C28—H28B	0.9600
C13—H13C	0.9600	C28—H28C	0.9600
C14—N3	1.4686 (14)	C29—N6	1.4682 (13)
C14—C15	1.5184 (16)	C29—C30	1.5161 (17)
C14—H14A	0.9700	C29—H29A	0.9700
C14—H14B	0.9700	C29—H29B	0.9700
C15—H15A	0.9600	C30—H30A	0.9600
C15—H15B	0.9600	C30—H30B	0.9600
C15—H15C	0.9600	C30—H30C	0.9600
			
N1—C1—C2	123.17 (10)	N4—C16—C17	123.29 (10)
N1—C1—C6	113.32 (9)	N4—C16—C21	113.76 (9)
C2—C1—C6	123.33 (9)	C17—C16—C21	122.86 (9)
C3—C2—C1	118.12 (10)	C18—C17—C16	118.14 (10)
C3—C2—H2	120.9	C18—C17—H17	120.9
C1—C2—H2	120.9	C16—C17—H17	120.9
C2—C3—C4	119.41 (10)	C19—C18—C17	119.22 (10)
C2—C3—H3	120.3	C19—C18—H18	120.4
C4—C3—H3	120.3	C17—C18—H18	120.4
C3—C4—C5	118.44 (10)	C18—C19—C20	118.61 (10)
C3—C4—H4	120.8	C18—C19—H19	120.7
C5—C4—H4	120.8	C20—C19—H19	120.7
N1—C5—C4	122.88 (10)	N4—C20—C19	122.84 (9)
N1—C5—C11	116.55 (9)	N4—C20—C26	116.66 (9)
C4—C5—C11	120.30 (10)	C19—C20—C26	120.35 (9)
O1—C6—N2	123.47 (10)	O3—C21—N5	123.73 (10)
O1—C6—C1	118.47 (9)	O3—C21—C16	119.16 (9)
N2—C6—C1	117.99 (9)	N5—C21—C16	117.09 (9)
N2—C7—C8	111.25 (9)	N5—C22—C23	111.66 (9)
N2—C7—H7A	109.4	N5—C22—H22A	109.3
C8—C7—H7A	109.4	C23—C22—H22A	109.3
N2—C7—H7B	109.4	N5—C22—H22B	109.3
C8—C7—H7B	109.4	C23—C22—H22B	109.3
H7A—C7—H7B	108.0	H22A—C22—H22B	107.9
C7—C8—H8A	109.5	C22—C23—H23A	109.5
C7—C8—H8B	109.5	C22—C23—H23B	109.5
H8A—C8—H8B	109.5	H23A—C23—H23B	109.5
C7—C8—H8C	109.5	C22—C23—H23C	109.5
H8A—C8—H8C	109.5	H23A—C23—H23C	109.5
H8B—C8—H8C	109.5	H23B—C23—H23C	109.5
N2—C9—C10	111.82 (9)	N5—C24—C25	111.75 (9)
N2—C9—H9A	109.3	N5—C24—H24A	109.3
C10—C9—H9A	109.3	C25—C24—H24A	109.3
N2—C9—H9B	109.3	N5—C24—H24B	109.3
C10—C9—H9B	109.3	C25—C24—H24B	109.3
H9A—C9—H9B	107.9	H24A—C24—H24B	107.9
C9—C10—H10A	109.5	C24—C25—H25A	109.5
C9—C10—H10B	109.5	C24—C25—H25B	109.5
H10A—C10—H10B	109.5	H25A—C25—H25B	109.5
C9—C10—H10C	109.5	C24—C25—H25C	109.5
H10A—C10—H10C	109.5	H25A—C25—H25C	109.5
H10B—C10—H10C	109.5	H25B—C25—H25C	109.5
O2—C11—N3	123.47 (10)	O4—C26—N6	123.44 (9)
O2—C11—C5	118.13 (9)	O4—C26—C20	118.43 (9)
N3—C11—C5	118.34 (9)	N6—C26—C20	118.11 (9)
N3—C12—C13	113.75 (9)	N6—C27—C28	113.31 (10)
N3—C12—H12A	108.8	N6—C27—H27A	108.9
C13—C12—H12A	108.8	C28—C27—H27A	108.9
N3—C12—H12B	108.8	N6—C27—H27B	108.9
C13—C12—H12B	108.8	C28—C27—H27B	108.9
H12A—C12—H12B	107.7	H27A—C27—H27B	107.7
C12—C13—H13A	109.5	C27—C28—H28A	109.5
C12—C13—H13B	109.5	C27—C28—H28B	109.5
H13A—C13—H13B	109.5	H28A—C28—H28B	109.5
C12—C13—H13C	109.5	C27—C28—H28C	109.5
H13A—C13—H13C	109.5	H28A—C28—H28C	109.5
H13B—C13—H13C	109.5	H28B—C28—H28C	109.5
N3—C14—C15	113.57 (9)	N6—C29—C30	113.32 (9)
N3—C14—H14A	108.9	N6—C29—H29A	108.9
C15—C14—H14A	108.9	C30—C29—H29A	108.9
N3—C14—H14B	108.9	N6—C29—H29B	108.9
C15—C14—H14B	108.9	C30—C29—H29B	108.9
H14A—C14—H14B	107.7	H29A—C29—H29B	107.7
C14—C15—H15A	109.5	C29—C30—H30A	109.5
C14—C15—H15B	109.5	C29—C30—H30B	109.5
H15A—C15—H15B	109.5	H30A—C30—H30B	109.5
C14—C15—H15C	109.5	C29—C30—H30C	109.5
H15A—C15—H15C	109.5	H30A—C30—H30C	109.5
H15B—C15—H15C	109.5	H30B—C30—H30C	109.5
C5—N1—C1	117.90 (9)	C20—N4—C16	117.81 (9)
C6—N2—C7	118.62 (9)	C21—N5—C24	124.13 (9)
C6—N2—C9	124.19 (9)	C21—N5—C22	119.09 (9)
C7—N2—C9	116.98 (9)	C24—N5—C22	116.76 (8)
C11—N3—C14	118.41 (9)	C26—N6—C29	118.26 (9)
C11—N3—C12	124.65 (9)	C26—N6—C27	125.00 (9)
C14—N3—C12	116.35 (9)	C29—N6—C27	116.11 (8)

### Hydrogen-bond geometry (Å, °)

**Table:**

Cg2 is the centroid of the N4/C16–C20 ring.

**Table:**

*D*—H···*A*	*D*—H	H···*A*	*D*···*A*	*D*—H···*A*
C3—H3···N4^i^	0.93	2.49	3.397 (1)	164
C9—H9B···O4^ii^	0.97	2.52	3.485 (1)	175
C12—H12B···O3^ii^	0.97	2.59	3.531 (1)	164
C18—H18···N1	0.93	2.52	3.427 (1)	166
C24—H24A···O2^iii^	0.97	2.46	3.417 (1)	169
C27—H27A···O1^iii^	0.97	2.54	3.496 (1)	168
C13—H13B···Cg(2)^ii^	0.97	2.97	3.726 (1)	139

Symmetry codes: (i) *x*, *y*−1, *z*; (ii) −*x*+1, −*y*+1, −*z*+1; (iii) −*x*+1, −*y*+1, −*z*+2.
